# Measuring out-of-field dose to the hippocampus in common radiotherapy indications

**DOI:** 10.1186/s13014-023-02242-3

**Published:** 2023-04-07

**Authors:** Hendrik Auerbach, Yvonne Dzierma, Michaela Schürmann, Christian Rübe, Claudia E. Rübe

**Affiliations:** Department of Radiation Oncology, Saarland University Medical Center, Homburg, Saar Germany

**Keywords:** Radiotherapy, Hippocampus, Out-of-field-dose, Thermoluminescent dosimetry

## Abstract

**Background:**

The high susceptibility of the hippocampus region to radiation injury is likely the causal factor of neurocognitive dysfunctions after exposure to ionizing radiation. Repetitive exposures with even low doses have been shown to impact adult neurogenesis and induce neuroinflammation. We address the question whether the out-of-field doses during radiotherapy of common tumour entities may pose a risk for the neuronal stem cell compartment in the hippocampus.

**Methods:**

The dose to the hippocampus was determined for a single fraction according to different treatment plans for the selected tumor entities: Point dose measurements were performed in an anthropomorphic Alderson phantom and the out-of-field dose to the hippocampus was measured using thermoluminescence dosimeters.

**Results:**

For carcinomas in the head and neck region the dose exposure to the hippocampal region for a single fraction ranged from to 37.4 to 154.8 mGy. The hippocampal dose was clearly different for naso-, oro- and hypopharynx, with maximal values for nasopharynx carcinoma. In contrast, hippocampal dose levels for breast and prostate cancer ranged between 2.7 and 4.1 mGy, and therefore significantly exceeded the background irradiation level.

**Conclusion:**

The mean dose to hippocampus for treatment of carcinomas in the head and neck region is high enough to reduce neurocognitive functions. In addition, care must be taken regarding the out of field doses. The mean dose is mainly related to scattering effects, as is confirmed by the data from breast or prostate treatments, with a very different geometrical set-up but similar dosimetric results.

## Background

About 50% of all patients with cancer receive treatment with radiation therapy during the progression of their disease [1, 2]. According to estimations, radiation therapy contributes to about 40% of the curative treatment [3]. As an achievement of numerous technical and medical-oncological innovations over the past decades, overall survival has been improved for many indications, therefore avoiding treatment-related adverse effects has gained increasing priority. The understanding of the radiobiological effects and their consequences in radiation therapy has also witnessed rapid improvements. Recent preclinical studies have shown that even low doses of ionizing radiation affect adult neurogenesis in the hippocampal region and induce long-lasting neuroinflammation. Repeated exposures with doses of 100 mGy (0.1 Gy) have been shown to compromise the structural and functional integrity of hippocampal neurogenesis which has been associated with neurocognitive dysfunctions that affect learning and memory [4–7]. Numerous clinical studies have aimed to reduce the hippocampus dose in brain radiotherapy by hippocampus-avoiding dose distributions [8–12].

While a number of studies have assessed hippocampus dose in cranial irradiation, particularly for brain tumours [13, 14], only limited data are available on the realistic dose to the hippocampus by radiotherapy for other tumour entities. In particular, new radiotherapy techniques such as intensity-modulated radiotherapy (IMRT) and volume-modulated radiotherapy (VMAT) provide highly conformal dose distributions to the target volume, but at the cost of an increased volume exposed to low radiation doses. Furthermore, the more highly modulated radiotherapy techniques generally require more monitor units to cover the target volume, which results in higher scattered and leakage doses. These actual dose exposures are not easily assessed during the treatment planning process, since the planning computer tomography (CT) rarely extends to the hippocampus for tumour entities located outside the brain or head-and-neck region—given the ALARA principle, a larger cranial elongation of the CT scan is in most cases out of the question. Furthermore, current treatment planning systems (TPS) generally underestimate out-of-field doses, even if the planning CT was extended to the hippocampus region [15–17]. Several studies observed that with greater distance from the field edge, the degree of dose underestimation increases and approaches nearly 100% at farther distances [18, 19]. This may lead to serious underestimations of both secondary cancer risks and potential deterministic effects for affected organs. The possible reason for these TPS errors is thought to be the underestimation of scattered radiation (due to beam modifiers in the near field), leakage radiation and internal patient scattering at greater distances from the field edge [18].

The aim of the present study is hence to investigate the out-of-field exposure in the hippocampus region during fractionated radiotherapy of common tumor entities, i.e. carcinoma of the head-and-neck (H&N) region, breast and prostate cancer. These entities were chosen because they are in close distance to the hippocampus region (H&N carcinomas) or present the most common radiotherapy indications (breast and prostate cancer). To our knowledge, measurements of hippocampus dose in realistic treatment scenarios have not been presented in the literature so far for tumour localizations outside the brain or head-and-neck region. The research question of this study was to assess doses to the hippocampus in common radiotherapy cases and in the light of their potential hazard to the neural stem cell compartment within the hippocampus.

## Methods

Exemplary treatment plans were chosen from our in-house database of patients treated in our department (in total 6 plans for the H&N region with different distances to the hippocampus and respectively two plans for breast and prostate treatment). All patients received treatment at one of three beam-matched linear accelerators (two Siemens Artiste and one Siemens Oncor) with 160 MLC operating at 6 MV or 18 MV photon energy [20].

The H&N plans used 6 MV IMRT in a simultaneous integrated boost (SIB) techniques with 13 beams, with sum doses of 54.12, 59.40 and 69.96 Gy, respectively. In addition, the H&N plans were divided into three subgroups with respect to treatment of naso-, oro- and hypopharyngeal carcinomas, for the purpose of a detailed dose investigation in the vicinity of the out-of-field exposure. For breast cancer, a representative planning scenario from our department was chosen: a step-and-shoot IMRT SIB fractionation of 28 × 1800 mGy to the whole breast (sum dose of 50.40 Gy) and 28 × 2140 mGy to the boost volume (sum dose of 50.40 59.92 Gy). Furthermore, the two patients were selected for the measurements because their breast volumes corresponded relatively well to the volume of the small (350 cc) and large (1200 cc) breast attachments for the anthropomorphic phantom available at our department. For prostate and prostate bed treatment, two 11-beam step-and-shoot IMRT plans using 6 MV photons were used, one with a planning target volume (PTV) dose of 66 Gy in single fraction doses of 2000 mGy, the other with a double SIB to 60 Gy total dose.

All treatment planning was performed with Philips Pinnacle TPS (Koninklijke Philips N.V., Amsterdam, The Netherlands) using the collapsed cone convolution (CCC) algorithm. Treatment plans were inversely optimized using direct machine parameter optimization (DMPO) to achieve acceptable target coverage while minimizing dose to local avoidance structures. For plan evaluation, we used our in-house dose-volume histogram (DVH) criteria based on the Quantec guidelines [21]. All treatment plan characteristics like the dose prescriptions as well as the number of beams and segments of the treatment plans are presented in Table 1.

**Table 1:** Treatment plan characteristics

Treatment plan		Beams	Segments	Dose per fraction [mGy]	Number of fractions	Total dose [Gy]
H&N (PTV + 2SIBs)	Naso I	13	49	2120, 1800, 1640	33	69.96, 59.40, 54.12
	Naso II	13	54	2120, 1800, 1640	33	69.96, 59.40, 54.12
	Oro I	13	41	2120, 1800, 1640	33	69.96, 59.40, 54.12
	Oro II	13	47	2120, 1800, 1640	33	69.96, 59.40, 54.12
	Hypo I	13	40	2120, 1800, 1640	33	69.96, 59.40, 54.12
	Hypo II	13	44	2120, 1800, 1640	33	69.96, 59.40, 54.12
Mamma left (PTV + SIB)	Ma large	7	45	2140, 1800	28	59.92, 50.40
	Ma small	6	29	2140, 1800	28	59.92, 50.40
Prostate (PTV + 2SIBs; PTV)	Prostate I	11	39	3000, 1800, 1640	20	60.00, 36.00, 32.80
	Prostate II	11	35	2000	33	66.00

**Fig. 1:**
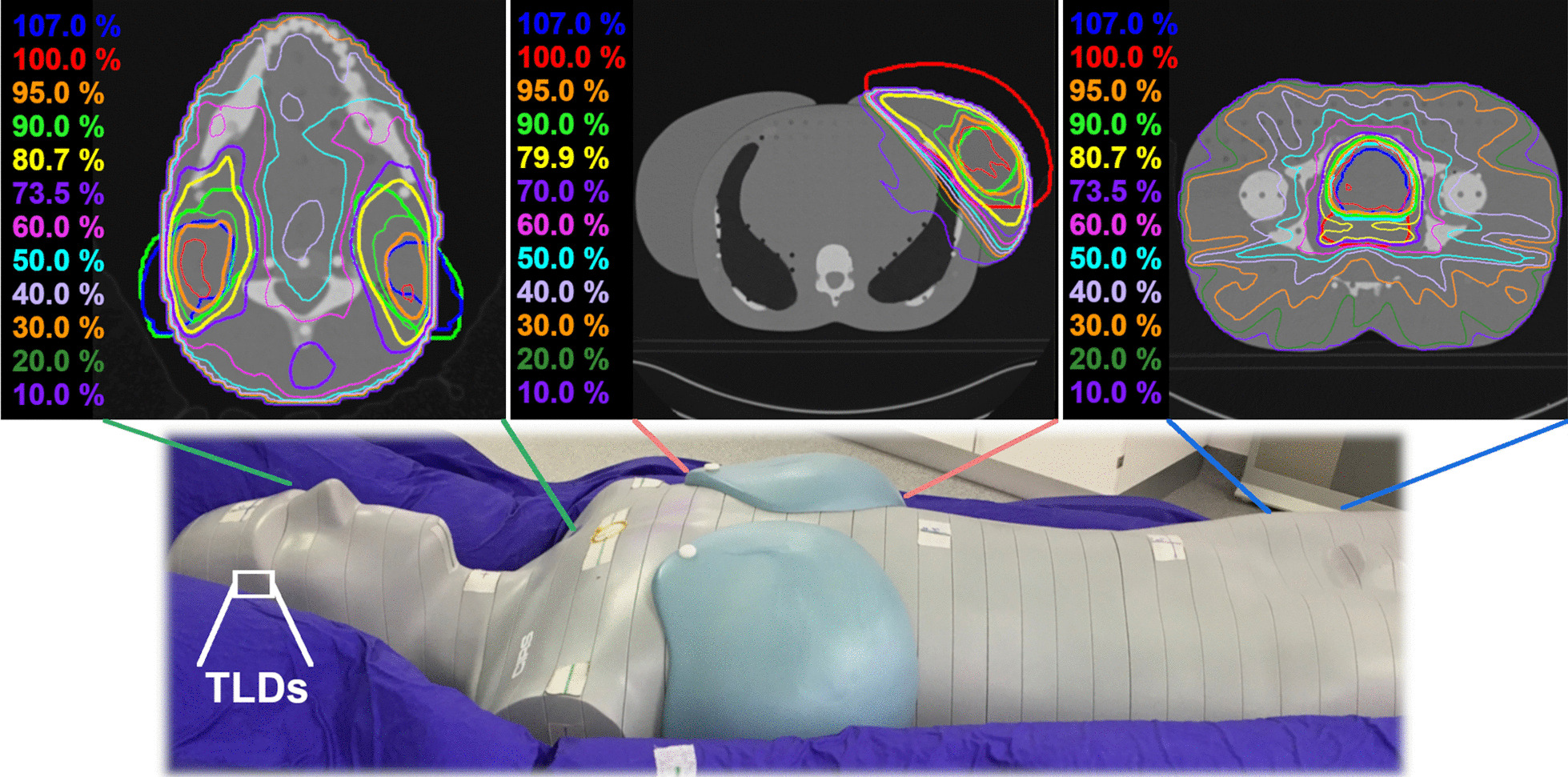
TLD position and the chosen anatomical region for the treatments in the Alderson phantom

The point dose measurements were carried out in an anthropomorphic Alderson phantom (CIRS, Norfolk, USA) (Fig. 1). In order to ensure a reproducible positioning, the phantom was placed in a BlueBag (Elekta, Stockholm, Sweden) vacuum cushion. A planning CT of the Alderson phantom was made and rigidly registered to the selected treatment plans in the TPS, so that the anatomical location of the isocenter could be adequately defined and the phantom precisely aligned according to the room lasers. The dose distributions and contours mapped to the Alderson phantom are shown in Fig. 1. All plans were exported for irradiation to the Artiste linac.

**Fig. 2:**
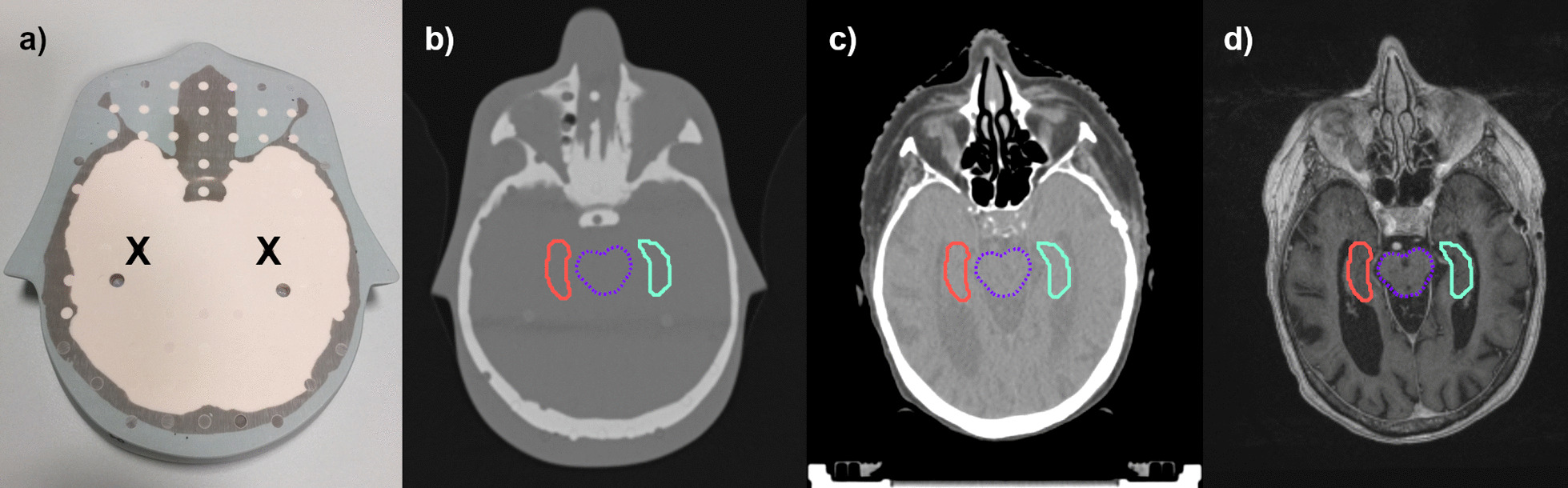
Positions of TLD measurements at the expected position of the hippocampus inside the Alderson phantom (**a**). The localization of the TLDs inside the Alderson head was determined (**b**) by registering the Alderson CT data set to a real patient CT (**c**) and MRI head scan (**d**), on which the hippocampus was contoured

For all three irradiations areas, the out-of-field dose to the hippocampus for a single treatment fraction was measured three times inside the phantom using thermoluminescent dosimeters (TLD 100H, Thermo Fisher Scientific, Waltham, MA, USA) and is given in mGy. The cumulative dose (in Gy) was calculated by the number of fractions and the measured out-of-field dose for a single treatment fraction. The localization of the hippocampus inside the Alderson head was determined by registering the Alderson CT data set to a real patient CT and MRI head scan, on which the hippocampus was contoured as described detailed elsewhere [8, 22, 23]. Each section of the phantom is drilled in a 1.5 cm × 1.5 cm grid pattern of 5 mm diameter through holes and all the drillings are filled with a solid plug equivalent to the surrounding tissue. For the measurement, the TLDs were placed as close to the realistic hippocampus positions as achievable with the manufactured drillings (Fig. 2). A packed TLD was placed at each of the two positions and replaced at each measurement. In addition, 3–10 TLDs were deposited outside the irradiation room to determine the background irradiation during the experiment. The average signal of the background was subtracted from the dose values of all dosimeters from the batch. All TLDs were calibrated using a Sr90/Y90 TLD irradiator (Thermo Electron 2210, Thermo Fisher Scientific, USA) to obtain a charge-to-dose calibration factor for each chip. Heating, reading and annealing were performed with a Harshaw TLD 5500 reader (Thermo Fisher Scientific, USA) using the vendor-recommended time–temperature protocol with the following three steps: at first the preheating step, where the TLDs are heated up to 145 °C for 5 s; subsequently the acquisition at 10 °C/s with a maximum temperature of 260 °C for 23 1/3 s, and finally annealing with hot nitrogen gas at 260 °C for 20 s. A detailed description of the calibration and measurement settings for the TLDs could be found in earlier publications of the authors [24, 25]. For the H&N treatments the TLD measurements are also compared with the calculated dose ranges from the TPS, which are read out as point dose values at the upper and lower end of the drillings in which the TLDs were placed in the phantom.

## Results

The point doses of the left and right hippocampus for a single fraction are listed in Table 2 and range between 2.709 ± 0.237 and 155.797 ± 30.586 mGy. For comparison, the last column in Table 2 shows the averages of the background dose (0.009–0.162 mGy), which is clearly below the measured dose in the hippocampus region.

**Table 2:** Doses and standard errors at the hippocampus after irradiation of different irradiation areas

Irradiation area			Prescription	Single fraction [mGy]			Multi fractions [Gy]	Background (single fraction [mGy])
				Mean dose ± 1 std. err. + (min.–max.)		Mean dose ± 1 std. err	Dose range of subgroup	
				Left	Right			
H&N (PTV + 2SIBs)	Naso I		33 × 2120 33 × 1800 33 × 1640	155.797 ± 30.586 (121.451–195.741)	153.881 ± 15.106 (136.751–173.501)	154.839 ± 24.140	2.816–6.459	0.009
	Naso II		33 × 2120 33 × 1800 33 × 1640	110.911 ± 8.077 (99.713–118.461)	129.148 ± 38.344 (85.342–178.731)	120.030 ± 29.170		
	Oro I		33 × 2120 33 × 1800 33 × 1640	51.041 ± 1.591 (48.981–52.854)	53.265 ± 5.572 (46.478–60.125)	52.153 ± 4.245	1.534–2.158	0.009
	Oro II		33 × 2120 33 × 1800 33 × 1640	56.656 ± 6.230 (51.307–65.394)	55.650 ± 5.599 (51.163–63.544)	56.153 ± 5.944		
	Hypo I		33 × 2120 33 × 1800 33 × 1640	59.671 ± 5.763 (51.964–65.820)	61.682 ± 1.189 (60.094–62.955)	60.676 ± 4.281	1.078–2.172	0.162
	Hypo II		33 × 2120 33 × 1800 33 × 1640	37.391 ± 1.054 (35.915–38.307)	37.487 ± 3.686 (32.677–41.631)	37.439 ± 2.711		
Mamma left (PTV + SIB)	Ma large		28 × 2140 28 × 1800	3.391 ± 0.092 (3.270–3.491)	3.240 ± 0.176 (2.993–3.392)	3.315 ± 0.159	0.084–0.131	0.013
	Ma small		28 × 2140 28 × 1800	4.438 ± 0.212 (4.156–4.666)	3.801 ± 0.141 (3.639–3.983)	4.119 ± 0.366		
Prostate (PTV + 2SIBs)	Prostate I		20 × 3000 20 × 1800 20 × 1640	3.628 ± 0.164 (3.397–3.758)	3.863 ± 0.106 (3.731–3.991)	3.746 ± 0.181	0.068–0.080	0.013
Prostate (PTV)	Prostate II		33 × 2000	2.709 ± 0.237 (2.409–2.989)	2.714 ± 0.118 (2.553–2.831)	2.711 ± 0.187	0.079–0.099	0.013

**Fig. 3:**
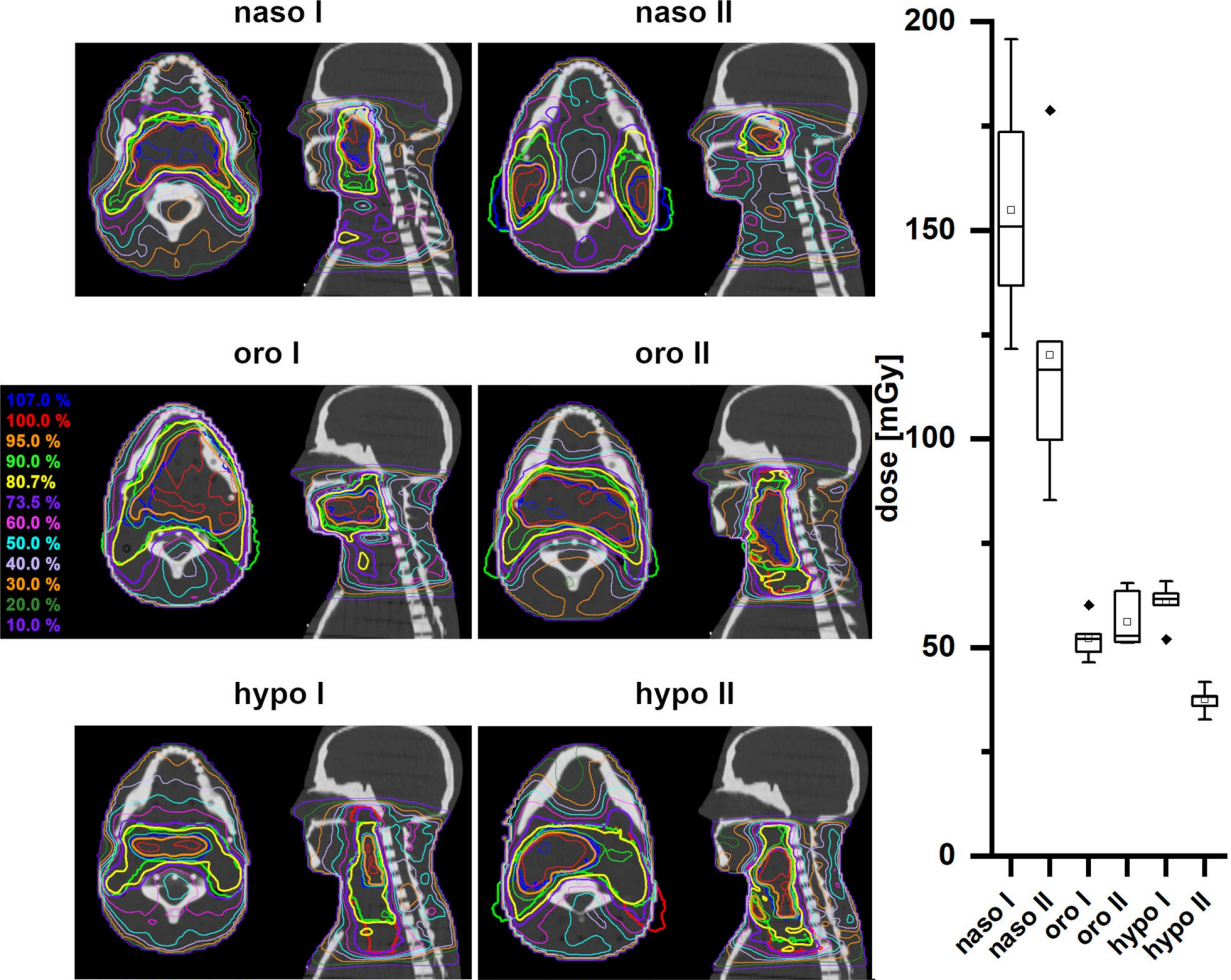
Left panel: Dose distributions of naso-, oro- and hypopharyngeal cancer treatment plans (PTV (red), SIB1 (green), SIB2 (blue)) with sum doses of 69.96 Gy. Right panel: Box plots of the measured doses of a single fraction for the different treatment plans of the different H&N regions

As expected, the dose in the hippocampus region was highest for the treatment of tumors in the H&N region (Fig. 3). All treatment plans for H&N tumors have a prescribed dose of 2120 mGy per fraction. In the treatment plans for nasopharyngeal carcinomas (subgroup: naso I and II) with the shortest cranial distance to the hippocampus, the average hippocampus doses are 154.839 ± 24.140 and 120.030 ± 29.170 mGy. Correspondingly, for oropharyngeal carcinomas, the hippocampus receives an average fraction dose of 52.153 ± 4.245 and 56.153 ± 5.944 mGy in the treatment plans (oro I and II). In the treatment plans for hypopharyngeal carcinomas (hypo I and II) with the longest cranial distance to the hippocampal region, the average hippocampus doses are 60.676 ± 4.281 and 37.439 ± 2.711 mGy. The dose differences of approximately 75% are mainly due to the different cranial extension of the different PTVs.

**Fig. 4:**
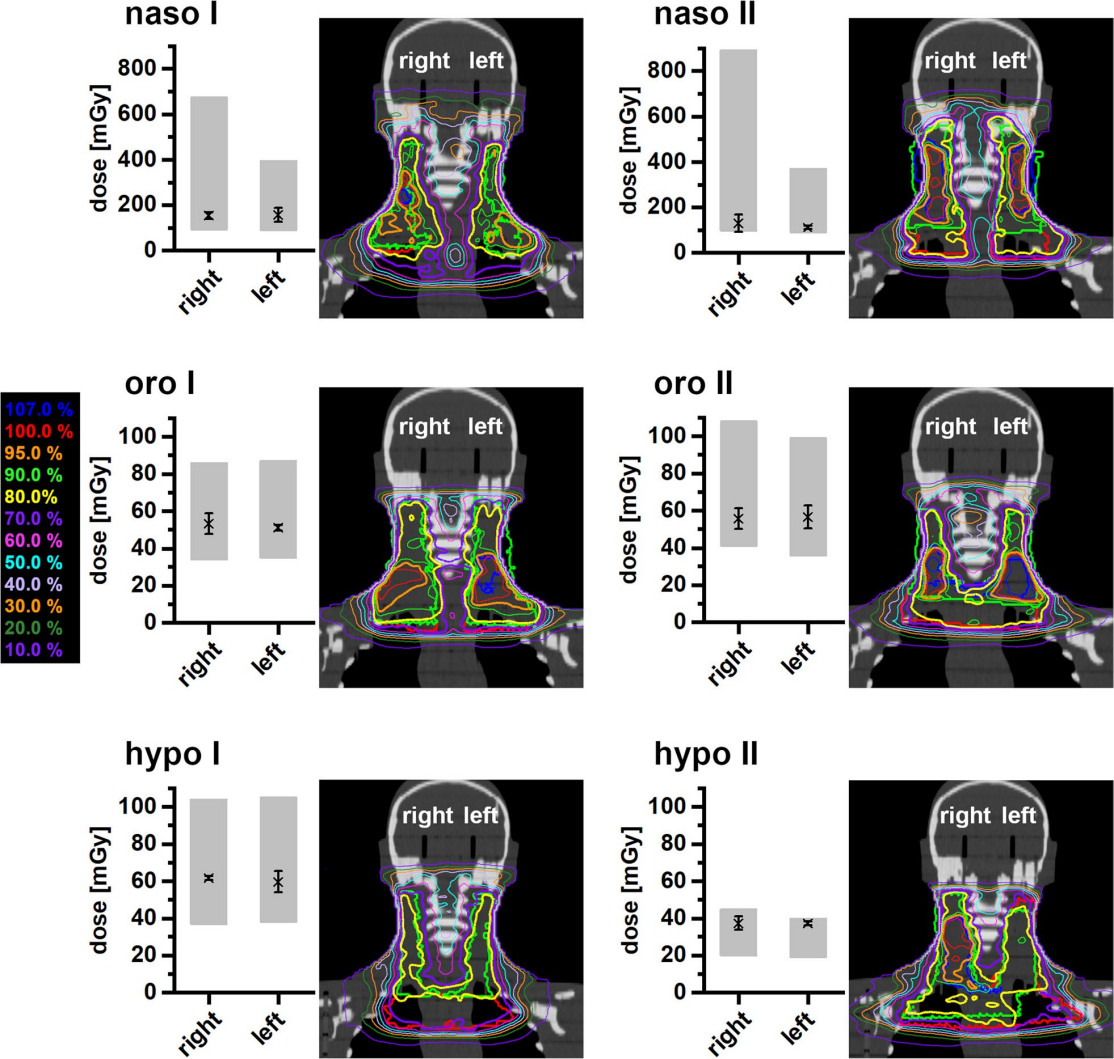
Measured mean dose at the left and right hippocampus position during the treatment of naso-, oro- and hypopharyngeal carcinomas (marked with X). The grey bars represent the dose limits determined from the treatment planning system for the drillings in the Alderson phantom in which the TLDs were positioned

Comparing the measured doses to the hippocampus for the H&N treatment plans with the calculated dose from the TPS, good agreement is observed when taking into account the fact that some uncertainty regarding the exact localization of the TLDs inside the phantom drillings exists (Fig. 4). The point doses calculated at the lower edge of the drilling fall closest to the upper limit of the PTV and hence cranial field edge; they are therefore larger than the measured values. Conversely, the point doses at the upper edge of the drilling present the minimum expected dose values, and are accordingly smaller than the measured doses.

**Fig. 5:**
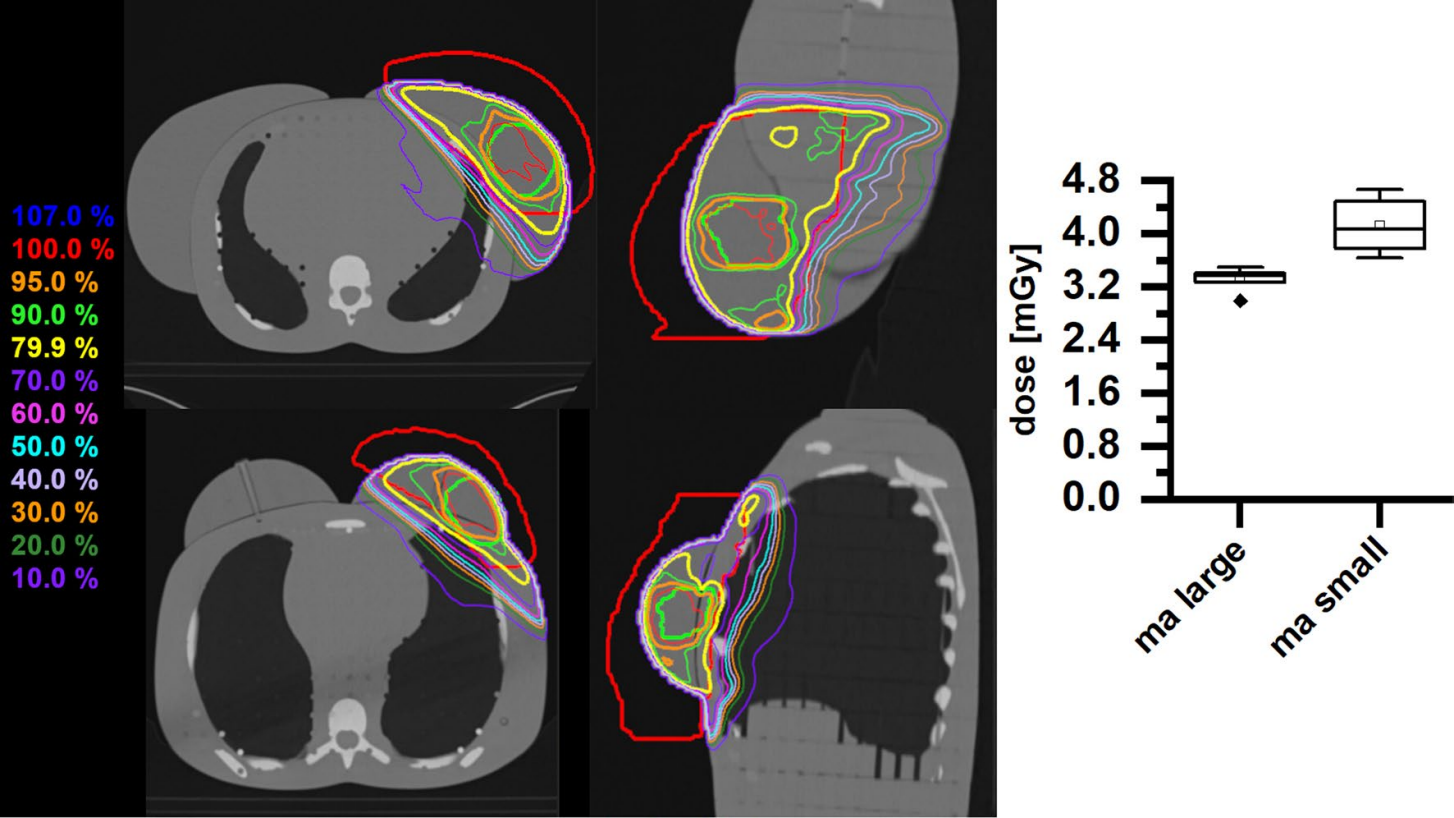
Left panel: dose distributions of breast treatment plans (PTV (red), SIB (green)) with sum doses of 59.92 Gy (upper trace—large breast; lower trace—small breast). Right panel: box plot of the measured doses of a single fraction for the different breast treatment plans

The two exemplary selected IMRT plans for breast carcinomas each for a large-volume and small-volume breast (ma large and ma small) differ only slightly with respect to the dose at the hippocampus per fraction (Fig. 5). In both cases, the measurement yields an average dose (3.315 ± 0.159 mGy and 4.119 ± 0.366 mGy), below 11% of the average dose measured for the treatments in the H&N region. While the IMRT breast treatment plan with the small breast attachment does show a somewhat higher hippocampus dose as compared with the plan with the large breast attachment, this result may be caused by the different distance of the PTV edge from the hippocampus in the two patient cases.

**Fig. 6:**
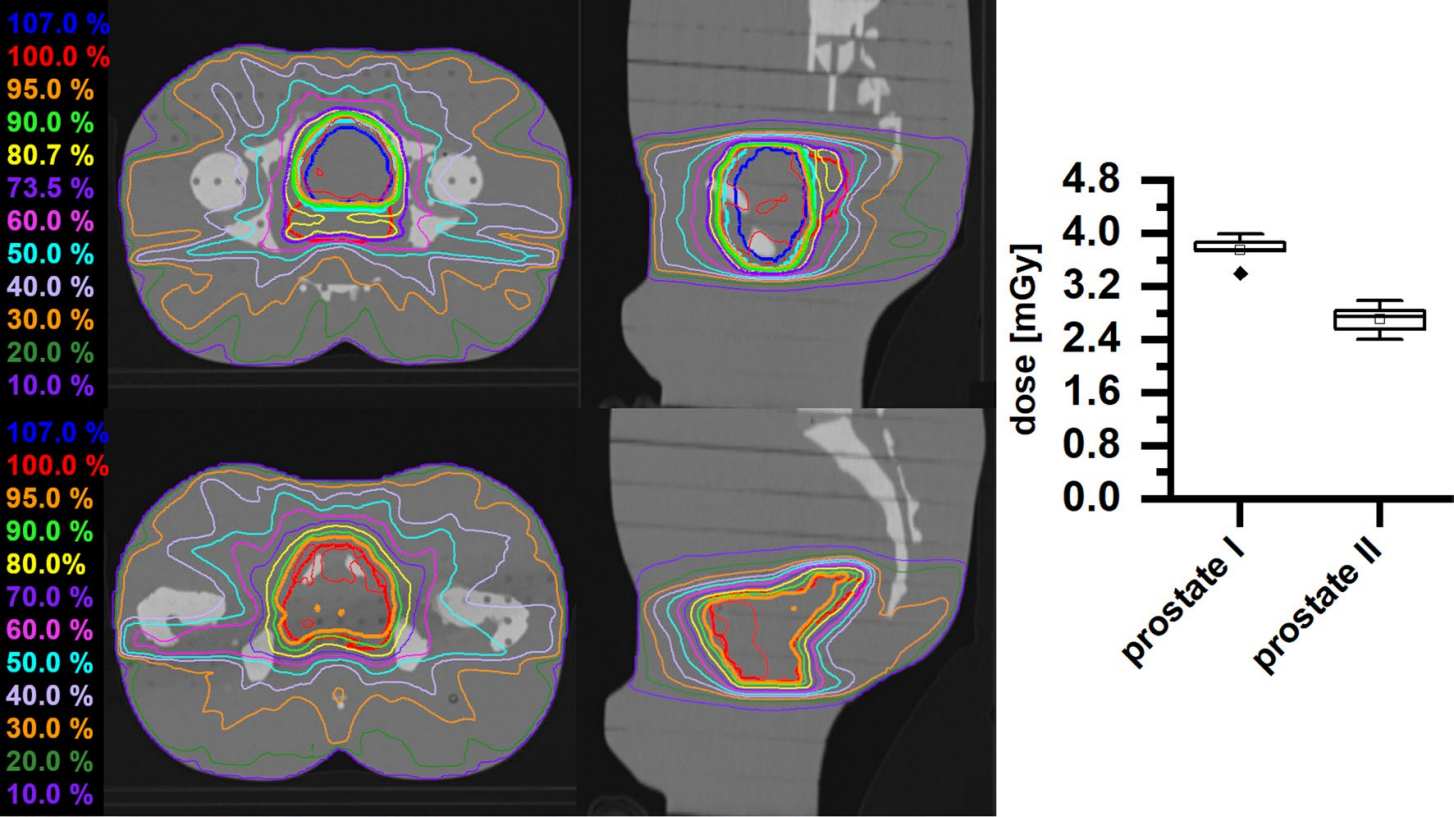
Left panel: dose distributions of a prostate treatment plan (PTV (red), SIB1 (green), SIB2 (blue)) with a sum dose of 60.00 Gy (upper trace) and a prostate bed treatment plan (PTV (red)) with a sum dose of 66.00 Gy (lower trace). Right panel: box plot of the measured doses of a single fraction for the different prostate treatment plans

Irradiation of prostate PTVs (prostate I and II), although located considerably more caudally than the breast treatment volume, yields only slightly lower dose values to the hippocampus (average 3.746 ± 0.181 mGy and 2.711 ± 0.187 mGy, Fig. 6).

## Discussion

### Overview of results and comparison with TPS calculations

**Fig. 7:**
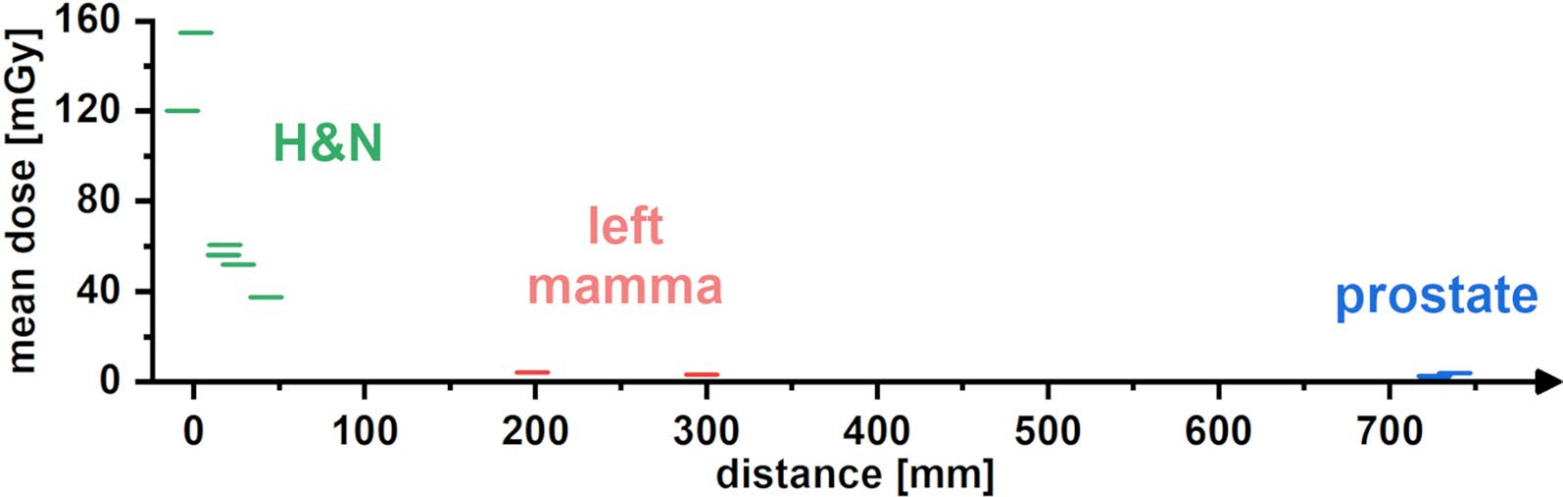
Measured mean dose for the TLDs placed at the two hippocampus positions of a single fraction concerning the different treatment regions. The mean dose decreases with increasing cranial distance

An overview of all irradiation areas together with the measured mean hippocampus doses is shown in Fig. 7. The boxplots show that with increasing distance between the hippocampus and the PTV, the mean doses to the hippocampus region decreases. In particular, this correlation is clearly visible for the treatment of carcinomas in the H&N region. The mean dose exposure of the hippocampus ranges from 120 to 160 mGy for nasopharyngeal carcinomas and decreases to the values of 40–60 mGy for the treatment of hypopharyngeal carcinomas. Due to the short distances between the PTV margin and hippocampal region, the hippocampus is still in the near-field region to the radiation field [26]. Here, dose exposure to the hippocampus is primarily caused by scattered radiation from the accelerator head, from the patient, and from leakage. In this range of distances to the hippocampus, the averaged dose values of the point dose experiments in the H&N region are within the error limits between the point doses calculated by the planning system at the lower and upper edge of the drilling for all three subgroups (Fig. 4). While our study showed a good agreement between estimated and measured dose values, other authors have observed that dose calculations from the treatment planning system, even in the near field region, can considerable over- or underestimate the real values [27, 28]. Considering realistic brain, lung and breast treatment plans calculated with the Phillips Pinnacle CCC algorithm, Huang et al. [18] reported an underestimation of the dose by the TPS of 30% or more, even at 3–4 cm from the field edge. A possible explanation for our very good agreement between the calculated and experimentally determined dose values might be the used calculation algorithm in combination with machine and patient parameters like e.g. the irradiation angles of the scenario. The quality of the commissioning of the treatment planning system also plays an important role.

As the distance between PTV and hippocampal region increases, the mean dose to the hippocampus further decreases to values between 3 and 4 mGy for treatment of breast and prostate cancer and is significantly larger than the background (see Fig. 7). In this context, the determined dose values in the out-of-field hippocampus region are almost completely caused by the accelerator head and patient scattering and leakage radiation, whereby their relative contributions may depend on the distance and planning technique (for instance, 3D conformal radiotherapy vs. IMRT or VMAT [26, 29]). Determination of the corresponding dose in the planning system is not realistic in this out-of-field region, where dose discrepancies increase and can reach 100% or more, as the TPS dose values approach zero [18, 30].

### Previous studies on hippocampus dose

Several studies have investigated the hippocampus dose during radiotherapy of H&N cancer patients. All mean dose values of the hippocampus presented below are calculated values from the TPS and show a relative wide variability (e.g. Gulliford et al. [31, 32]). For a collective of 10 patients with nasopharyngeal cancer treated by IMRT with a total PTV dose of 70 Gy (D95%), Khodayari et al. [14] reported mean hippocampus doses of 30.27 Gy (range 19.08–47.99 Gy). Somewhat smaller values were observed for nasopharyngeal carcinoma treatment by Lei Shi et al. [33], with mean doses of 18.29 ± 9.1 Gy, 21.3 ± 12.0 Gy and 3.2 ± 2.0 Gy for left and right hippocampal head and right pulvinar, respectively, and by Sun Zong-Wen et al. (average dose of 11.5 ± 9.8 Gy and 10.1 ± 6.0 Gy to the left and right hippocampus, respectively) [13]. Similarly, Sharma et al. observed mean doses of 14.1 and 12.9 Gy for the treatment of sinonasal carcinoma [34]. Chia-Ju Chen et al. report a mean hippocampus dose of 6.89 ± 0.73 Gy, again for nasopharyngeal carcinoma patients [35]. For general H&N cancer treatment, Olsson et al. estimated radiation doses to the hippocampus in the range of 1.5–9.3 Gy [36], which compares to our measured point doses of 2.816–6.459 Gy for the nasopharyngeal carcinoma cases, when scaled for a complete treatment regime of 33 fractions. Evidently, there is a large variability of results depending on the cranial extension of the PTV and hence the proximity of the hippocampus to the cranial field edge. Furthermore, some optimization potential exists for hippocampus sparing in nasopharyngeal carcinoma radiotherapy, as has been pointed out by Han et al. (reduction of hippocampus mean dose from 24.1 Gy to 14.1 Gy [37]) and Gu et al. (reduction from 15.2 to 9.0 Gy [38]).

For target volumes located far from the brain, only few studies have investigated hippocampus dose exposure. Ghasemi-Jangjoo & Ghiasi present a Monte-Carlo study of out-of-field doses from a 18 MV prostate treatment plan, finding a photon dose to the brain [39]. In the present study, two prostate treatment plans of 60 Gy and 66 Gy total dose were analyzed, resulting in hippocampus doses of 0.068–0.080 Gy and 0.079–0.099 Gy, respectively. The prescriptions used in our study would scale to 0.112 Gy and 0.123 Gy estimated by Ghasemi-Jangioo & Ghiasi, which is good agreement, considering that they did not specify the hippocampus, but rather the brain in general. Our own previous measurements of scattered doses to the eye lens in prostate treatment with different flat and flattening-filter-free treatment techniques [40, 41] for a single 2000 mGy-fraction dose amount to 0.320–3.941 mGy, which would correspond to 9.600–118.230 mGy for a treatment regime up to 60 Gy total dose. These results are comparable to our present data for a similar, though not identical, scenario.

Recent preclinical studies have shown that fractionated irradiation with even low doses (100 mGy) leads to morphological and functional alterations of the stem/progenitor cell niche in the hippocampus. Repetitive radiation-induced injury to neuronal lineages and accessory glia cells causes stem cell niche degradation with progressive neuronal loss. Injury-related processes initiated soon after repetitive low-dose irradiation may synergistically alter the supporting structure integrity and signaling microenvironment and trigger long-lasting neuroinflammation. Collectively, radiation-induced damage of the hippocampal region even after repetitive low-dose irradiation drives complex pathophysiological processes that can lead to permanent cognitive decline [4–7, 42]. The dose values measured for tumour treatment in the H&N region (approximately 50–150 mGy per fraction in our study) fall into the dose range where biological effects have been shown to occur. For tumours located considerably farther from the brain, the dose to the hippocampus is of the order of a few mGy per fraction. In this regime, biological effects on the hippocampus have not been investigated so far, so the relevance of these doses is yet unclear. However, the summation dose over the whole treatment regime may amount to almost 100 mGy even for these scenarios, so that cellular effects cannot a priori be excluded.

## Conclusions

We present point dose measurements of the hippocampus in an anthropomorphic Alderson phantom during the irradiation of carcinoma in the H&N region as well as breast and prostate cancer. The measured dose confirm that as the distance between the hippocampus and the planning target volume increases, the mean dose decreases. In the H&N region the TLD measurements in the Alderson phantom agree well with the calculated TPS dose ranges and are therefore within the error limits between the dose limits from the TPS. Furthermore, the determined dose values for the H&N region are in the dose range, where impairments of the structural and functional integrity of hippocampal neurogenesis have been shown to occur.

## Data Availability

All datasets are available upon reasonable request.

## Abbreviations

CCC: Collapsed cone convolution
CT: Computer tomography
DMPO: Direct machine parameter optimization
DVH: Dose-volume histogram
H&N: Head-and-neck
IMRT: Intensity-modulated radiotherapy
PTV: Planning target volume
SIB: Simultaneous integrated boost
TLD: Thermoluminescent dosimeters
TPS: Treatment planning systems
VMAT: Volume-modulated radiotherapy
